# Markers for sepsis diagnosis in the forensic setting: state of the art

**DOI:** 10.3325/cmj.2014.55.103

**Published:** 2014-04

**Authors:** Cristian Palmiere, Marc Augsburger

**Affiliations:** University Center of Legal Medicine, Lausanne, Switzerland

## Abstract

Reliable diagnoses of sepsis remain challenging in forensic pathology routine despite improved methods of sample collection and extensive biochemical and immunohistochemical investigations. Macroscopic findings may be elusive and have an infectious or non-infectious origin. Blood culture results can be difficult to interpret due to postmortem contamination or bacterial translocation. Lastly, peripheral and cardiac blood may be unavailable during autopsy. Procalcitonin, C-reactive protein, and interleukin-6 can be measured in biological fluids collected during autopsy and may be used as in clinical practice for diagnostic purposes. However, concentrations of these parameters may be increased due to etiologies other than bacterial infections, indicating that a combination of biomarkers could more effectively discriminate non-infectious from infectious inflammations. In this article, we propose a review of the literature pertaining to the diagnostic performance of classical and novel biomarkers of inflammation and bacterial infection in the forensic setting.

Worldwide, sepsis and its sequels are still a common cause of acute illness and death in patients with community-acquired and nosocomial infections (1). Multiple organ dysfunction syndrome (MODS) is common in critical cases of severe sepsis and a primary cause of death (2). The American College of Chest Physicians and the Society of Critical Care Medicine Consensus Conference (Northbrook, IL, USA; August 1991) defined sepsis as a systemic inflammation response caused by infection (1,3,4). However, in the early stages of the process, the source of infection may be unclear and the related systemic response indistinguishable from non-infectious disease. Consequently, diagnosis may be missed or delayed, posing a serious concern since strong evidence associates early treatment with greater clinical success (5,6). At present, there is no ideal, clinical gold standard for the diagnosis of sepsis, as microbiology is not sensitive enough and laboratory tests unspecific for use as a reference standard (1,2,5).

Bacteremia is identified in only a portion of patients with sepsis, also depending on previous antibiotic treatment. Culture-negative patients accounted for percentages ranging from 25% to 48% of all septic cases in a series of clinical studies performed in North America, Europe, and Asia (1,7).

Early clinical signs, such as fever, tachycardia, and leukocitosis, are usually unspecific and overlap with signs of systemic inflammatory response syndrome (SIRS) of non-infectious origin. More specific signs of sepsis, such as arterial hypotension, thrombocytopenia, and increased lactate concentration, often indicate progression to organ dysfunction (1,8).

Though C-reactive protein (CRP) and procalcitonin are currently used as clinical indicators of inflammation and infection, several other biochemical markers have been investigated for their ability to detect sepsis in an early, reversible phase (1,3). Nonetheless, identification of an ideal biomarker (or panel of biomarkers) capable of making a clear distinction between sepsis and SIRS is imperative (2).

Reliable diagnoses of sepsis remain challenging in the forensic field, despite improved methods of collecting blood and tissue samples for postmortem bacteriology and extensive research in biochemical and immunohistochemical investigations. There are a myriad of reasons for this difficulty in diagnosis, such as the fact that forensic pathologists rarely have full access to medical records before autopsy is performed. Furthermore, macroscopic findings (myocardial ischemia, pulmonary edema, hypoxic liver damage, mesenteric ischemia, gastrointestinal hemorrhages, spleen infarction, kidney ischemia, and brain edema) and histological observations may be elusive or non-specific and have an infectious or non-infectious origin. In addition, blood culture results can be difficult to interpret due to contamination during sampling procedures or bacterial translocation. Postmortem samples might also prove insufficient, unavailable, or absent during autopsy, especially in infant autopsy. Procalcitonin, CRP, and interleukin-6 can be measured in biological fluids collected during autopsy and may be used as in clinical practice for diagnostic purposes. However, the concentrations of these parameters can be increased due to etiologies other than bacterial infections. Hence, as in the clinical field, in recent years other laboratory parameters have been investigated in order to define the most suitable biomarker (or combination of biomarkers) that might more effectively discriminate non-infectious from infectious inflammations (9-15).

The identification of reliable markers of sepsis in the forensic setting is, however, more difficult than in the clinical field. Indeed, biochemical profiles after death may show considerable variations consequent to various factors including survival time, molecule leakage from necrotic or damaged cells due to rapid cell membrane breakdown, molecule redistribution dependent on concentration gradients, and molecule denaturation. All these factors therefore limit the application of postmortem biochemistry to relatively stable markers and only some, specific biological fluids (16-18). The aim of this article is to propose a review of the literature pertaining to the diagnostic performance of classical and novel biomarkers of sepsis in forensic pathology routine.

## Procalcitonin

High serum procalcitonin concentrations were first described in children with severe bacterial infections by Assicot et al (19), and were suggested as a specific marker for bacterial infection. Procalcitonin, the precursor of the hormone calcitonin, is a glycoprotein consisting of 116 amino acids with a molecular weight of 13 kDa. Gene products transcribed from the CALC-I gene (located on the short arm of the human chromosome 11) represent a large array of related proteins including calcitonin gene-related peptide, amyllin, adrenomedullin, and calcitonin (calcitonin family) that are restricted to neuroendocrine cells of the thyroid under normal metabolic conditions. Only one of these peptides, procalcitonin, seems to play a pivotal role in the host response to microbial infections. Procalcitonin is produced from the common precursor, pre-procalcitonin, which consists of 141 amino acid residues by removal of 25 amino acids from the N-terminus. Procalcitonin is made up of a centrally placed calcitonin (a 32-amino acid peptide) and two flanking peptides, N-terminal region (a 57-amino acid peptide), and katalcin (a 21-amino acid peptide). Regular enzymatic processing and further cleavage in the C-cells of the thyroid gland result in the production of mature procalcitonin. In the absence of infection, the extra-thyroid transcription of the CALC-I gene is suppressed. However, during bacterial infections, pre-procalcitonin mRNA is ubiquitously expressed in various extra-thyroid neuroendocrine tissues and non-neuroendocrine parenchymal cells throughout the body. Consequently, under septic circumstances, procalcitonin is released into the bloodstream by a continuous constitutive pathway. Elevated concentrations of procalcitonin during severe bacterial infections are commonly observed in patients who had already undergone total thyroidectomy. In patients with sepsis, non-neuroendocrine parenchymal cells are stimulated to produce and secrete large amounts of procalcitonin. However, these lack the post-translational capacity of neuroendocrine cells to biosynthesize mature calcitonin hormone from procalcitonin. A significant increase in calcitonin during sepsis therefore rarely occurs. Though hepatocytes may produce procalcitonin, it is still debated whether the liver and splancnic area produce procalcitonin during infections (3,8,20-25).

Systemic activation of immunocompetent cells in response to microorganisms or microbial endotoxin induces inflammatory cytokine release at the infection site. This is the pivotal mechanism in the pathogenesis of the sepsis cascade. Even though procalcitonin is not produced by circulating blood cells, its synthesis seems to be closely dependent on the cytokines involved in initiating the inflammatory cascade. Procalcitonin can be detected after 3-4 hours in response to bacterial endotoxins and peaks at 6-8 hours with a half-life of approximately 24-30 hours (8,20-22).

In healthy individuals, all the procalcitonin produced in C-cells is converted to calcitonin, so that circulating procalcitonin concentrations are below detection levels of 0.1 ng/mL (0.1 μg/L). In patients with sepsis, procalcitonin concentration may increase up to 5000 to 10 000 times with calcitonin still in the reference range. No enzymes in blood can break down the procalcitonin circulating molecule (8,20,22).

The physiologic role of procalcitonin is not yet completely understood. Bacterial endotoxins are the most potent stimulators of procalcitonin release, though gram-positive infections may also induce its production. Apart from bacterial infections, various situations of tissue damage in non-infected patients such as prolonged cardiogenic shock, major surgery, severe trauma, or burns, may induce increases in blood procalcitonin concentrations. However, blood levels observed under these conditions are usually not as high as those in patients with severe sepsis or septic shock. Hence, in cases of trauma or extensive surgery, increased level persistence as well as secondary procalcitonin increases at a later time may herald the onset of infectious complications. Local viral infections do not induce increased procalcitonin levels, whereas systemic viral infections may determine levels as high as those noted in bacterial infections. Systemic fungal infections may be characterized by increased procalcitonin levels, though concentrations tend to be lower than those in patients with bacterial infections. Conversely, infections by the malaria parasite often lead to very high serum procalcitonin levels (8,20,23).

Procalcitonin determination for the postmortem diagnosis of sepsis in the realm of forensic pathology was originally proposed by Tsokos et al (26). These authors measured procalcitonin concentrations in postmortem serum obtained from septic and control cases and compared antemortem and postmortem procalcitonin values in septic cases. They observed increased procalcitonin levels in all septic cases and normal concentrations in most control cases. They also observed a postmortem decrease in procalcitonin values compared to antemortem levels, suggesting that the postmortem measurement of this marker seems reasonable until at least 140 hours after death. These authors concluded that increased procalcitonin levels could be considered a reliable diagnostic tool for the postmortem diagnosis of sepsis. Furthermore, they proposed at least two positive postmortem procalcitonin values at different postmortem intervals in order to better estimate the most probable procalcitonin level at the time of death by using linear regression analysis.

Vitreous, pericardial, and cerebrospinal fluid procalcitonin levels in septic and control cases were investigated by Schrag et al (10,27), who reported promising results with vitreous and pericardial fluid procalcitonin concentrations in septic cases. Bode-Jänisch et al (28) measured procalcitonin concentrations in postmortem serum obtained from septic and control cases and compared postmortem levels to antemortem procalcitonin concentrations when available. The results confirmed the stability of procalcitonin in postmortem samples and the reliability of this biomarker in the postmortem diagnosis of bacterial sepsis.

## C-reactive protein

CRP, named for its capacity to precipitate the somatic C-polysaccharide of Streptococcus pneumoniae, was the first, acute-phase protein to be described. It is a systemic marker of inflammation and tissue damage, widely used in the diagnosis and management of various clinical conditions. The acute-phase response comprises the nonspecific physiological and biochemical responses of endothermic animals to most forms of tissue damage, infection, inflammation, and malignant neoplasia. In particular, the synthesis of numerous proteins is rapidly up-regulated, mainly in hepatocytes, under the control of cytokines originating at the site of tissue damage. The human CRP molecule is composed of five identical non-glycosylated polypeptide subunits, each containing 206 amino acid residues. In healthy young adults, the median concentration of CRP is 0.8 mg/L, the 90th percentile is 3.0 mg/L, and the 99th percentile is 10 mg/L, but, following an acute-phase stimulus, values may increase to more than 500 mg/L, hence 10 000 times. Plasma CRP is produced only by hepatocytes, predominantly under transcriptional control of IL-6, although other sites of local CRP synthesis and possibly secretion have been suggested. De novo hepatic synthesis starts very rapidly after a single stimulus, with concentrations rising above 5 mg/L by about 4 to 6 hours and peaking around 24 to 48 hours. CRP plasma half-life is about 19 hours and is constant under all conditions of health and disease, so that the sole determinant of circulating CRP concentration is the synthesis rate, which thus directly reflects the intensity of the pathological process(es) stimulating its production (3,29-31).

CRP levels remain elevated during the acute response phase. When the stimulus for increased production completely ceases, the circulating CRP concentration falls rapidly and returns to within reference values when tissue damage is resolved. Due to its rapid response, short half-life, and high magnitude of increase, CRP is a useful indicator of the acute phase response in several situations. These may include infections (bacterial, viral, fungal, and mycobacterial), inflammatory diseases, necrosis (myocardial infarction, pancreatitis), trauma, and malignancy. In most, though not all, diseases, the circulating value of CRP reflects ongoing inflammation and/or tissue damage much more accurately than do other laboratory parameters of the acute-phase response. Acute-phase CRP values show no diurnal variation and are unaffected by eating. Liver failure impairs CRP production, but no other intercurrent pathologies and very few drugs reduce CRP values unless they also affect the underlying pathology providing the acute-phase stimulus. CRP concentration is thus a very useful, nonspecific biochemical marker of inflammation as well as an important measurement that may contribute to determining disease severity and progression (30,31).

In the forensic field, the diagnostic application of CRP determination was originally proposed by Laurier et al (32). These authors measured CRP values in postmortem serum and pericardial fluid in a series of 26 forensic autopsies that were selected based on agony duration (long and short agonies). The results of this study showed statistically significant increases in CRP levels in both postmortem serum and pericardial fluid in long agonies. The authors attributed this result to agonal pericarditis possibly resulting from agonal myocardial necrosis.

Further studies focusing on CRP levels in postmortem samples have been performed by several other research teams (10,27,29,33-38). Uhlin-Hansen (33), Astrup and Thomsen (37), and Tsokos et al (34) compared antemortem and postmortem CRP levels and observed increased concentrations of this marker in septic cases, reflecting the existence of ongoing inflammatory processes at the time of death. These authors also found that antemortem CRP levels were higher than postmortem concentrations, likely suggesting molecule proteolysis due to decompositional changes along with irrelevant postmortem molecule release from hepatocytes in the early postmortem period. Maeda et al (35) found low CRP levels in some infantile and elderly cases of pneumonia that were postulated as age-dependent, low inflammatory responses. Astrup and Thomsen (37) observed that CRP levels in postmortem serum stored at 5°C were stable for several weeks. CRP levels measured in liver samples (collected from the central part of the right lobe), though less stable, correlated well with postmortem serum samples.

## Interleukin-6

Interleukin-6 (IL-6) is a multifunctional, proinflammatory cytokine with pleiotropic expressions produced by a wide variety of cell types (leukocytes, fibroblasts, and endothelial cells) in the early phase of inflammation. It is a 26 kDa protein that modulates a variety of functions and has a key role in the acute-phase inflammatory response, being the primary determinant of hepatic CRP production. In addition to this role, IL-6 is important in specific immunologic response development, including activated B cell differentiation, culminating in the production of immunoglobulin (34,39-44).

IL-6 is normally not detected in the serum of healthy young individuals unless there is trauma, infection, or some other stress. Under these circumstances, IL-6 is rapidly expressed and contributes to a cascade of events typical of inflammation. These include leukocytosis, lymphocyte activation, and acute-phase protein synthesis as well as a general catabolic shift in metabolic pathways (39).

IL-6 plays a central role in the pathogenesis of sepsis. Numerous clinical studies have demonstrated consistently increased IL-6 values in both adult and pediatric septic populations. Serum levels above 1000 pg/mL have been shown to predict sepsis-related death in adult patients (3).

Tsokos et al (34) investigated IL-6 levels in the postmortem serum of a series of sepsis and control cases, comparing antemortem and postmortem levels. They observed high (>1.000 pg/mL) values in most individuals included in the sepsis group as well as a notable increase in IL-6 concentration in postmortem serum compared with IL-6 levels in antemortem samples. According to the authors, this increase correlated well with the interval after death and suggested molecule release from cells storing IL-6 due to autolysis and decompositional changes.

## Soluble interleukin-2 receptor

Interleukin-2 (IL-2) is secreted from activated T cells in several immunologic processes and is the major growth factor for T-lymphocytes. IL-2 acts by binding with a membrane IL-2 receptor (IL-2R, CD25), which is widely expressed by many leukocytes that include activated B cells, monocytes, eosinophil granulocytes, and natural killer cells. Early in the activation process, T cells express IL-2R, which consists of three subunits (α, β, γ) encoded by different genes. The combination of IL-2Rβ and γ can bind IL-2. However, the expression of all three subunits is required for the high affinity state. In situations where a high expression of membrane IL-2R occurs, the subunit α (IL-2Rα, sIL-2R or sCD25) is shed from the cell surface by proteolytic cleavage and released into the circulation. sIL-2R release was found to be proportional to its membrane bound-expression, and its determination was therefore proposed as a useful, indirect marker of T- cell activation in several immunologic situations including various chronic autoimmune diseases, neoplastic disorders, acute graft-vs-host disease, chronic liver diseases, and sepsis (45-48).

Reichelt et al (49) investigated sIL-2R in the postmortem serum of a series of sepsis and control cases, comparing antemortem and postmortem levels. They observed increased (>1.000 U/mL) levels in all individuals included in the sepsis group and values below the reference limit in most control cases. Antemortem levels were generally higher than postmortem concentrations. Moreover, using linear regression analysis, sIL-2R levels calculated for the time of death correlated well with the levels measured in antemortem samples.

## Lipopolysaccharide binding protein

Innate immunity is the first line of defense against microbial infections. Host organism responses are activated when microbial components are recognized by a variety of pathogen sensors, stimulating the host defense effector system by rapidly triggering proinflammatory processes. Among microbial components, lipopolysaccharide (LPS), lipo-oligosaccharides (LOS), and their bioactive portion, lipodisaccharide lipid A are commonly defined as endotoxins. These are potent immune response stimulants and even small differences in LPS structure can have a great influence on host immune responses. The induction of inflammatory responses by endotoxins is achieved by the coordinated, sequential action of four principal endotoxin-binding proteins: LBP (LPS-binding protein), CD14, toll-like receptor 4 (TLR4), and MD-2 (myeloid differentiation protein), herein further discussed (50).

LBP was first described in 1990. It is a 58-kDa glycoprotein mainly synthesized in the liver, which is released into circulation as a type I acute-phase reactant. Reference plasma level ranges from 5 to 15 µg/mL. LBP levels peak shortly after bacteremia or endotoxemia, and remain increased for up to 72 hours later (8,51).

LBP interacts with endotoxin-rich bacterial membranes and purified endotoxin aggregates, catalyzing endotoxin monomer extraction and transfer to CD14, which in turn transfers endotoxin monomers to MD-2 and to MD-2–TLR4 complex. The transfer of LPS from CD14 to MD-2, coupled with the association of MD-2 to TLR4, is required for downstream signaling and initiation of the intracellular signal cascade that culminates in transcription factor translocation to the nucleus and cytokine biosynthesis. Some of these, specifically IL-6, in turn induce the synthesis of acute-phase proteins in the liver (50-55).

Forensic use of LBP was investigated by Reichelt et al (49) in a series of sepsis-related deaths and control subjects. LBP levels were above 10 µg/mL (reference limit in healthy subjects) in serum samples obtained prior to death and postmortem serum samples obtained during autopsy in all septic cases. A notable decrease in LBP levels was observed in the sepsis group, with concentrations still measurable up to 48 hours after death. A less marked decrease in LBP levels was also noted in the control cases. According to the authors, the decrease in postmortem serum LBP levels correlated well with the interval after death in both studied groups and suggested molecule proteolysis due to decompositional changes along with irrelevant postmortem molecule release from hepatocytes in the early postmortem period. The authors concluded that increased LBP levels could be considered a reliable diagnostic tool for the postmortem diagnosis of sepsis. They proposed at least two measurements at different postmortem intervals in order to better estimate the most probable LBP levels at the time of death by using linear regression analysis.

Augsburger et al (56) compared procalcitonin and LBP values in postmortem serum and pericardial fluid in a series of sepsis-related cases and control subjects. They observed increased (>10 µg/mL) values in most septic cases and reference (<10 µg/mL) levels in most control cases, thereby confirming a high diagnostic accuracy of LBP in identifying sepsis-related deaths. No associations were found between postmortem serum and pericardial fluid LBP levels in either the septic or control cases.

## sCD14-ST

CD14, the high-affinity receptor for LPS-LBP complexes, is a 55-kDa glycosylphosphatidylinositol-anchored protein lacking a cytoplasmic domain. CD14 can only bind LPS in the presence of LBP and, although LPS is considered its main ligand, CD14 also recognizes other microbial constituents, including the proteoglycans of Gram-positive bacteria. CD14 is constitutively expressed in most innate immune response cells and exists either in an anchored membrane form (mCD14) or in a circulating soluble form (sCD14). The latter is a 43-53 kD glycoprotein that derives from either protease-mediated membrane CD14 shedding or liver synthesis as a type II acute-phase reactant. During inflammation, plasma protease activity generates soluble CD14 fragments: one of these is a 13-kDa truncated N-terminal fragment of 64 amino acid residues called sCD14 subtype (sCD14-ST) or presepsin (50-55,57-63).

By facilitating binding to the CD14 cell membrane molecules, LBP enhances the sensitivity of monocytes and granulocytes to LPS, whereas the soluble form of CD14 mediates LPS activation of CD14-negative cells (53).

sCD14-ST is normally present in very low concentrations in the serum of healthy individuals and has recently been suggested as a marker for the diagnosis of sepsis. Indeed, preliminary studies have indicated that presepsin values significantly differ in healthy individuals, in patients with local infection, SIRS, sepsis or severe sepsis (57-63).

In the forensic setting, increased postmortem serum sCD14-ST levels have been found in a series of sepsis-related deaths using cutoff values ranging from 600 to 1200 pg/mL. Though postmortem serum sCD14-ST levels, individually considered, failed to provide better sensitivity and specificity than procalcitonin in detecting sepsis cases, the combined determination of procalcitonin and sCD14-ST in parallel was proposed as useful in improving the diagnostic performance of each biomarker individually considered in situations with elusive, preliminary findings (64).

## Soluble triggering receptor expressed on myeloid cells-1

Triggering receptor expressed on myeloid cells type 1 (TREM-1) is a recently discovered member of the immunoglobulin superfamily engaged as a cell membrane receptor on the myeloid cell family. TREM-1 expression on monocyte and macrophage surfaces was shown to be markedly up-regulated in human biological fluids as well as tissues infected by gram-positive and gram-negative bacteria. The functional significance of TREM-1 was discovered when it was observed that the blockade of TREM-1 signaling protects mice from the lethal effects of lipopolysaccharide-induced septic shock. Moreover, up-regulation of cell-surface TREM-1 expression was shown to result in marked plasma elevation of the soluble form of the molecule (sTREM-1) (65-71).

Increased plasma sTREM-1 values, individually considered or in association with other laboratory parameters, have been described in patients with bacterial infections and sepsis. Additionally, elevated broncho-alveolar lavage fluid sTREM-1 concentrations were found in patients with bacterial or fungal pneumonia and increased pleural fluid sTREM-1 levels in patients with infectious effusions (72-79). However, increased plasma sTREM-1 concentrations were also observed in patients with acute pancreatitis and non-infectious inflammations after traumatic lung contusion or pulmonary aspiration syndromes (80-82). In addition, the diagnostic and prognostic performances of sTREM-1 in sepsis and septic shock was reported to be variable, controversial, and even contradictory according to other clinical studies that reaffirmed the central role of traditional laboratory parameters, such as CRP, IL-6, and procalcitonin, in distinguishing sepsis from systemic inflammatory response syndrome (65).

In the forensic field, sTREM-1 levels were determined in postmortem serum, pericardial fluid, and urine in septic and control cases. Increased postmortem serum sTREM-1 levels (cutoff value 90 pg/mL) were observed in sepsis-related deaths, whereas most control cases had postmortem serum sTREM-1 levels lower than 90 pg/mL. Increased sTREM-1 values were also found in pericardial fluid and urine in septic cases. However, postmortem serum sTREM-1 levels did not seem to provide improved sensitivity and specificity compared to procalcitonin in detecting sepsis after death, indicating that the simultaneous assessment of both biomarkers could eventually help in clarifying specific situations characterized by elusive macroscopic, microscopic, and laboratory findings (9).

## Endocan

The vascular endothelium has been demonstrated as playing a critical role in the pathogenesis of sepsis by producing cytokines and chemotactic factors as well as expressing surface adhesion molecules that induce circulating leukocyte migration into tissues. Consequently, there is a strong, biological rationale for targeting markers of endothelial activation as biomarkers of sepsis (83,84).

In clinical practice, a large number of molecules secreted by the endothelial cells have been investigated as potential biomarkers for the early diagnosis of sepsis. These have included regulators of endothelial activation, adhesion molecules, as well as mediators of coagulation, permeability, and vasomotor tone (84).

Endocan (endothelial cell-specific molecule-1) is a soluble 50-kDa proteoglycan made up of a mature polypeptide of 165 amino acids and a single dermatan sulfate chain covalently linked to the serine residue at position 137. The molecule is naturally expressed by endothelial cells, is highly regulated in presence of proinflammatory cytokines and proangiogenic molecules and may be considered an accurate marker of endothelial activation. Endocan expression was associated with a growing number of pathological conditions characterized by neoangiogenesis and vascular growth. The molecule was found freely circulating at low levels in the serum of healthy subjects and overexpressed by several types of human tumors, particularly highly vascularized cancers (85-97).

Additionally, increased endocan levels were described in patients with sepsis, severe sepsis, and septic shock compared to healthy individuals, with concentrations related to the severity of the disease. Scherpereel et al (83) proposed a cutoff value of 1.2 ng/mL, which would provide the best sensitivity (82.50%) and specificity (100%) in identifying septic cases vs SIRS cases. Since endothelial injury is pivotal in the development of organ failure and shock in sepsis, an endothelial marker such as endocan might not only reflect the severity of the disease but also represent a promising diagnostic and prognostic marker of sepsis (8,84,98).

In the forensic setting, endocan levels have been determined in postmortem serum in septic and control cases. Simultaneous increases in both procalcitonin and endocan levels were identified in septic cases only. Endocan concentrations were low or undetectable in most control cases, irrespective of the postmortem interval, suggesting that the molecule is not systematically released into the bloodstream after death following the onset of decompositional changes. Conversely, concentrations over 1.0 ng/mL were observed only in control cases characterized by diffuse vascular injuries, presumably indicating endocan leakage in the bloodstream due to direct endothelial cell damage (99).

## Neopterin

Neopterin (D-erytro-1’,2’,3′-trihydroxypropylpterin), a biochemical product of the guanosine triphosphate pathway, was first isolated from human urine in 1965. The discovery of neopterin as a marker of T-cell activation dates back to the 1980s. The molecule is produced primarily in monocyte/macrophage and related cells when stimulated by interferon-γ released from activated T cells. Other cell types do not produce measurable amounts of neopterin following various stimuli. Therefore, the production of the molecule appears to be closely associated with cellular immune system activation. The biological function of neopterin is not completely clear. However, it has been demonstrated that neopterin has relations with nitric oxide synthesis and reactive oxygen metabolites. Based on this, it has been postulated that the molecule may be toxic for microorganisms and be part of the proinflammatory and cytocidal armature of activated human macrophages. The upper limit of the reference range is approximately 10 nmol/L ( = 2.5 ng/mL) (8,100-104).

High neopterin concentrations in serum and urine were shown to be a reliable indicator of the severity of viral (HIV), bacterial, protozoic, parasitic, or fungi-induced infections. Correlations between neopterin levels and disease states were also found for autoimmune disorders (rheumatoid arthritis, systemic lupus erythematosus, Crohn’s disease, and autoimmune thyroid diseases). Serum levels of neopterin were shown to be elevated in subjects with coronary and peripheral artery disease, with concentrations potentially predicting adverse cardiovascular events and heart failure development in patients with coronary artery disease, acute coronary syndromes, or severe peripheral artery disease. In various types of malignant diseases, increased neopterin concentrations were indicated as predictive in tumor progression, metastasis development and mortality. Although not produced by tumor cells themselves, increased neopterin concentrations most likely reflect the host-defense reaction elicited by the aggressiveness of the tumor. Furthermore, the monitoring of neopterin concentrations would also allow early detection of immunological and infectious complications in allograft recipients following kidney, heart, liver, lung, pancreas, and bone marrow transplants. Since elevated levels are found in infectious and non-infectious inflammations, the specificity of neopterin as a clinical marker of bacterial sepsis is, however, limited (101-104).

Forensic use of neopterin was investigated by Ishikawa et al (36) and Ambach et al (105,106). Postmortem serum neopterin levels over 500 nmol/L were observed in bacterial and viral infection-related cases as well as in delayed deaths due to trauma, thus confirming systemic inflammatory response syndromes and monocyte/macrophage activation.

## Conclusions

Many advances have been made in the identification of novel biomarkers for sepsis diagnosis. However, substantial discoveries are yet to be made in this field, both in clinical and forensic casework. Although more than 178 biomarkers have been identified, it remains controversial which of these is the most reliable for the diagnosis of sepsis. None of the currently available markers can be used to undoubtedly determine whether or not a patient is infected. Similarly, in the forensic field, none of the currently available biomarkers can be used to establish a definite diagnosis of sepsis-related death. These limits notwithstanding, at present procalcitonin represents the most reliable parameter for the postmortem diagnosis of sepsis.

Due to the complexity of the sepsis response, several authors have postulated that a combination of biomarkers, rather than a single laboratory parameter, might be more effective in order to obtain early and reliable diagnosis of sepsis in living patients. In our opinion, this same consideration should be applied to the postmortem field, where the diagnosis of sepsis is even more challenging.

We consider that it is not advisable to reach the diagnosis of sepsis-related death based on laboratory investigations only, especially when biochemical analyses are limited to a single parameter. Conversely, in our view, postmortem bacteriology and postmortem biochemistry must always be part of the diagnostic work-up and results must always be interpreted in context, with information from circumstantial data and medical records (when available), autopsy, histology, and, when feasible, neuropathology and immunohistochemistry (1-3,8,107,108).
